# Separation of *cis* and *trans* Isomers of Polyproline by FAIMS Mass Spectrometry

**DOI:** 10.1007/s13361-016-1482-1

**Published:** 2016-10-04

**Authors:** Andrew J. Creese, Helen J. Cooper

**Affiliations:** School of Biosciences, University of Birmingham, Edgbaston Birmingham, B15 2TT UK

**Keywords:** High field asymmetric waveform ion mobility spectrometry, Differential mobility spectrometry, Peptides, Polyproline

## Abstract

High field asymmetric waveform ion mobility spectrometry (FAIMS) is well-established as a tool for separating peptide isomers (sequence inversions and post-translationally modified localization variants). Here, we demonstrate the FAIMS is able to differentiate *cis* and *trans* isomers of polyproline. Polyproline assumes an all-*cis* conformation—the PPI helix—in 1-propanol, and an all-*trans* conformation—the PPII helix—in aqueous solutions. Differentiation of these conformers may be achieved both through use of a cylindrical FAIMS device and a miniaturized ultrahigh field planar FAIMS device.

**Electronic supplementary material:**

The online version of this article (doi:10.1007/s13361-016-1482-1) contains supplementary material, which is available to authorized users.

**Graphical Abstract:**
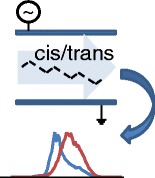
ᅟ

## Introduction

Proline is the only naturally occurring amino acid that can exist in both *cis* and *trans* conformations, unlike other amino acid residues, which favour *trans*. Several biological processes are associated with *cis*/*trans* isomerization of proline, including protein folding and cell signalling [1]. Counterman et al. [2] demonstrated the presence of *cis-* and *trans-* conformers of proline-containing peptide ions in the gas phase.

The peptide bradykinin (Arg-Pro-Pro-Gly-Phe-Ser-Pro-Phe-Arg) is perhaps the most studied proline-containing peptide with regard to *cis* and *trans* conformers. In a series of alanine substitution experiments, Pierson et al. showed that the proline residues were responsible for the multiple peaks observed in drift tube ion mobility spectra of triply protonated bradykinin ions [3]. Later work used collisional activation combined with ion mobility spectrometry to determine the energy barriers associated with interconversion of the various conformers [4]. Bradykinin has also been studied by FAIMS (high field asymmetric waveform ion mobility spectrometry [5, 6], also known as differential ion mobility, DMS). In 2001, Purves et al. combined FAIMS and H/D exchange and detected at least four conformers of doubly protonated bradykinin ions [7]. Papadopoulos et al. combined FAIMS with cold ion spectroscopy for the investigation of doubly charged bradykinin ion conformers, revealing two conformational families [8]. Multiple conformers of [M + 2H]^2+^ ions of bradykinin were also observed by Brown et al. in their evaluation of a miniaturised ultrahigh field FAIMS device [9]. Although not explicitly stated in these publications, in each case the multiple peaks observed in FAIMS spectra are likely due to *cis-* and *trans*-conformers. Shvartsburg et al. [10] considered the potential for *cis-* and *trans*- conformers in their FAIMS analyses of multiply phosphorylated peptides from the protein tau. Phosphorylation of specific threonine and serine residues within tau are known to influence *cis/trans* forms of adjacent proline residues.

**Figure 1:**
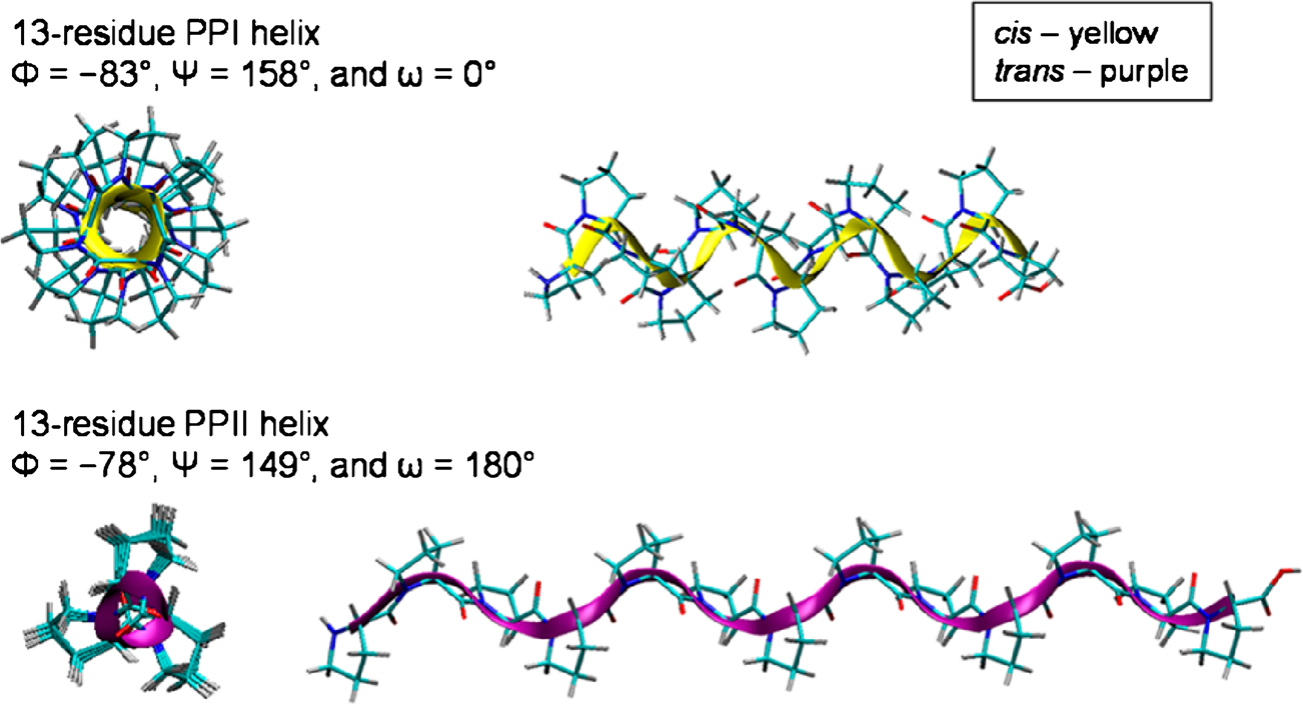
Representation of structures for polyproline-13 (Pro13): the all-*cis* PPI helix and the all-*trans* PPII helix. Reprinted with permission from [11]. Copyright 2014 American Chemical Society

In a series of recent papers, Clemmer and co-workers have studied the conformations of polyproline by drift tube ion mobility spectrometry [11–14]. Polyproline exists as two major conformers: the all-*cis* polyproline I (PPI) helix and the all-*trans* polyproline II (PPII) helix, see Figure 1. The former is favored in aliphatic alcohols, whereas the latter is favored in aqueous solutions. Clemmer and co-workers showed that by equilibrating solutions of polyproline-13 (Pro13) in 1-propanol and subsequent dilution in water, the conversion of PPI to PPII, via various *cis/trans* intermediates, could be followed by ion mobility spectrometry. At 5 °C, the conversion was complete by 800 min, and at 45 °C, the conversion was complete by 16 min.

Here, we investigate whether the conformers of Pro13 can be differentiated by FAIMS mass spectrometry. Solutions of Pro13 in 1-propanol, and aqueous solutions at various time points, were analyzed by two FAIMS devices: a ‘full-size’ cylindrical FAIMS device (with nitrogen as the carrier gas) and a miniaturized chip-based ultrahigh field planar device (with ambient air as the carrier gas). For each FAIMS device, a range of dispersion fields (DF) were employed. In each case, the compensation field (CF) corresponding to the maximum peak intensity [i.e., E_c(max)_], was calculated.

## Methods

The 13-mer polyproline peptide (Pro13) was purchased from Peptides and Elephants (Nuthetal, Germany) and resuspended in 1-propanol to a concentration of 160 μM. The solution was incubated at 37 °C for 72 h to ensure the peptide was in the PPI (all *cis*) conformation. As per the protocol by Shi et al. [11], the sample was diluted with water (J.T. Baker, Deventer, Holland) and acetic acid (Fisher Scientific, Bremen, Germany) to a final concentration of 16 μM in 1-propanol:water:acetic acid (10:88:2). The solutions were placed in a Thermomixer (Eppendorf, Hamburg, Germany) at 45 °C for 5, 10, 30, or 120 min, and then stored on ice until analysis. Results for the zero time point were obtained by electrospraying a 16 μM solution of Pro13 in 1-propanol and acetic acid (98:2).

### ESI-uFAIMS-MS

The sample was directly infused by use of a Triversa Nanomate (Advion Biosciences, Ithaca, NY, USA) with a spray voltage of 1.7 kV and a gas pressure of 0.3 psi. The sample tray of the Triversa Nanomate was cooled to 6 °C. The Owlstone uFAIMS device (Owlstone, Cambridge, UK) was coupled to an Orbitrap Elite mass spectrometer (Thermo Scientific) and was operated in 1D mode. Each sample was analyzed at six dispersion fields (30, 37.6, 45.1, 52.6, 60.1, 67.6 kV/cm, corresponding to 120, 150, 180, 210, 240, 270 Td). At each DF, the compensation field was ramped from −250 to 1000 V/cm (−1 to 4 Td) in 180 s. The mass spectrometer operated in MS mode and ions were detected in the Orbitrap. Data were recorded in the *m/z* range 150 to 2000 at a resolving power of 60,000 (at *m/z* 200). Automatic gain control (AGC) target was 1 × 10^6^ charges with a maximum fill time of 1000 ms. The ion transfer capillary was heated to 350 °C. Measurements were performed in triplicate on two separate days for a total of six repeats. All data were recorded using Xcalibur (Thermo Scientific, version 3.0).

### ESI-FAIMS-MS

The samples were also analyzed by use of a prototype cylindrical FAIMS device (supplied by Thermo Scientific) with electrode gap width of 1.5 mm [15]. The samples were infused at a rate of 3 μL/min through a HESI II probe with the syringe wrapped in an ice blanket. The electrospray voltage was set to 4 kV and nitrogen sheath gas was employed at 2 arbitrary units (~0.6 L/min) to aid desolvation. Each sample was analyzed at three dispersion fields (−20, −26.7, and −33.3 kV/cm, corresponding to dispersion voltages of −3, −4, and −5 kV). The FAIMS carrier gas was 100% N_2_ at flow rate 2 L/min. The inner and outer electrodes were 70 and 90 °C, respectively. At each DF, the compensation field was stepped from −30 to −400 V/cm in 2 V/cm increments. The mass spectrometer operated in MS mode and ions were detected in the Orbitrap. Data were recorded in the range *m/z* 150 to 2000 at a resolving power of 15,000 (at *m/z* 200). Automatic gain control (AGC) target was 1 × 10^6^ charges with a maximum fill time of 1000 ms. Measurements were performed in triplicate on two separate days for a total of six repeats. All data were recorded using Xcalibur (Thermo Scientific, version 3.0).

### Data Analysis

All Owlstone FAIMS data were analyzed using software developed in-house [16]. All Thermo FAIMS data were exported directly to Microsoft Excel for further processing. All plots shown are averages of the six repeats. To calculate E_c(max)_ (compensation field, E_c_, at maximum ion transmission) averaged data were analyzed in Matlab using Peakfit.m (http://terpconnect.umd.edu/~toh/spectrum/InteractivePeakFitter.htm). Between 1 and 4 Gaussian peaks were fitted to the data to give an error of <2% with the fewest number of peaks. Values stated correspond to the maximum of the largest fitted peak. Errors quoted correspond to one standard deviation.

## Results and Discussion

The electrospray mass spectra obtained for Pro13 in 1-propanol (t = 0 min) and in aqueous (t = 120 min) solution are shown in Supplementary Figure 1. Peaks corresponding to the singly and doubly charged peptide ions are observed in mass spectrum obtained from propanol, whereas only the doubly charged ion is observed from aqueous solution. This observation is similar to that seen by Clemmer and co-workers for the polyproline peptide Pro7 [12]. In their work, the singly charged species was exclusively observed for the PPI conformation, arising from protonation at the N-terminus; and the doubly charged species was exclusively observed for the PPII conformation, arising from protonation at the N-terminus and a backbone nitrogen. In the present work, we have focused on the separation of the doubly charged species of Pro13 by FAIMS.

The work by Clemmer and co-workers [11] revealed that at 45 °C, the dominant conformer of doubly protonated ions of Pro13 in 1-propanol is all *cis* (CCCCCCCCCCCC; C = *cis*), with an additional conformer of approximately 50% abundance corresponding to TTCCCCCCCCCC (T = *trans*). At t = t = 120 min, the only conformers are TTTTTTTTTTTT (dominant), TTTTTTTTCCCC (~70% abundance), and TTTTTTTTTTCT (~10% abundance). If it is the case that no conformational changes are induced by the FAIMS device, the above should describe the conformers present in the samples studied here. Any field heating by the FAIMS device that results in large changes in differential ion mobility will result in loss of the ion due to the self-cleaning mechanism. Ions experiencing field-induced conformational changes associated with small changes in differential ion mobility would still be transmitted [17, 18].

**Figure 2:**
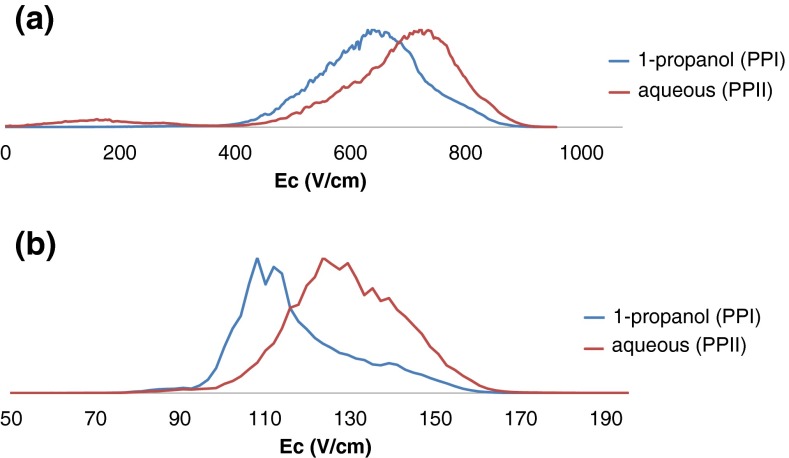
Separation of cis/trans isomers of Pro13 by FAIMS mass spectrometry. (**a**) FAIMS spectrum obtained by use of the Owlstone miniaturized ultrahigh field planar device at DF = 67.6 kV/cm (270 Td) with ambient air carrier gas. (**b**) FAIMS spectrum obtained by use of the Thermo cylindrical device at DF = 26.7 kV/cm (DV = −4000 V). Blue traces correspond to Pro13 in 1-propanol (t = 0 min) and red traces correspond to Pro12 after dilution in water (t = 120 min)

Figure 2a and Supplementary Figure 2 show FAIMS spectra obtained by use of the Owlstone uFAIMS device with ambient air as the carrier gas. The uFAIMS is a miniaturized device comprising interleaved planar electrodes. The small gap width enables the use of high electric fields without electrical breakdown of the carrier gas. The resolving power of the device, however, is low because of the short separation times. At each dispersion field and timepoint, a single broad peak was observed. The values for │E_c(max)_│ increase with increasing time (i.e., the lowest│E_c(max)_│is observed for the samples comprising PPI conformers and the highest for the sample comprising PPII conformers). The best separation, as determined by the greatest value of ΔE_c(max_,_),_ was achieved at DF = 67.6 kV/cm. The values for │E_c(max)_│ (i.e., the compensation field at the maximum peak intensity), were 651 ± 23 and 746 ± 15 V/cm for time points 0 and 120 min, respectively.

The results from the ‘full size’ cylindrical device, which employs lower dispersion fields, are shown in Figure 2b and Supplementary Figure 3. At DF = 20 kV/cm and 26.7 kV/cm, again, a single broad peak was observed at each time point, with │E_c(max)_│correlating with increased time (i.e., transition from PPI to PPII). At DF = 33.3 kV/cm, two peaks were observed at each time point. │E_c(max)_│ for the two peaks are ~210 and ~248 V/cm, although their relative abundances differ. That is, it was not possible to distinguish the samples comprising PPI and PPII conformers at DF = 33.3 kV/cm. The best separation was achieved at │DF│ = 26.7 kV/cm (DV = −4000 V): At t = 0 min (when the all-*cis* conformer dominates), │E_c(max)_│ was 112 ± 3 with peak width (FWHM) 20 V/cm; at t = 120 min (when the all-*trans* conformer dominates), │E_c(max)_│ was 132 ± 3 with peak width (FWHM) 34 V/cm.

## Conclusion

The results show that FAIMS analyses of samples comprising PPI and PPII conformers of Pro13 result in FAIMS peaks with differing values of │E_c(max)_│ (i.e., it is possible to differentiate *cis/trans* isomers of Pro13 with FAIMS). This result can be achieved both with a miniature planar FAIMS device and a ‘full-size’ cylindrical device.

## Electronic supplementary material

Below is the link to the electronic supplementary material.

ESM 1 (PPTX 466 kb)
