# Supplementary material for: Separation of cis and trans Isomers of Polyproline by FAIMS Mass Spectrometry
Source: J Am Soc Mass Spectrom. 2016 Oct 4;27(12):2071–4. doi: 10.1007/s13361-016-1482-1 (PMC5088216; doi:10.1007/s13361-016-1482-1)
Supplement: Supplementary file 1 — (PPTX 466 kb) [file 13361_2016_1482_MOESM1_ESM.pptx]

## Slide 1
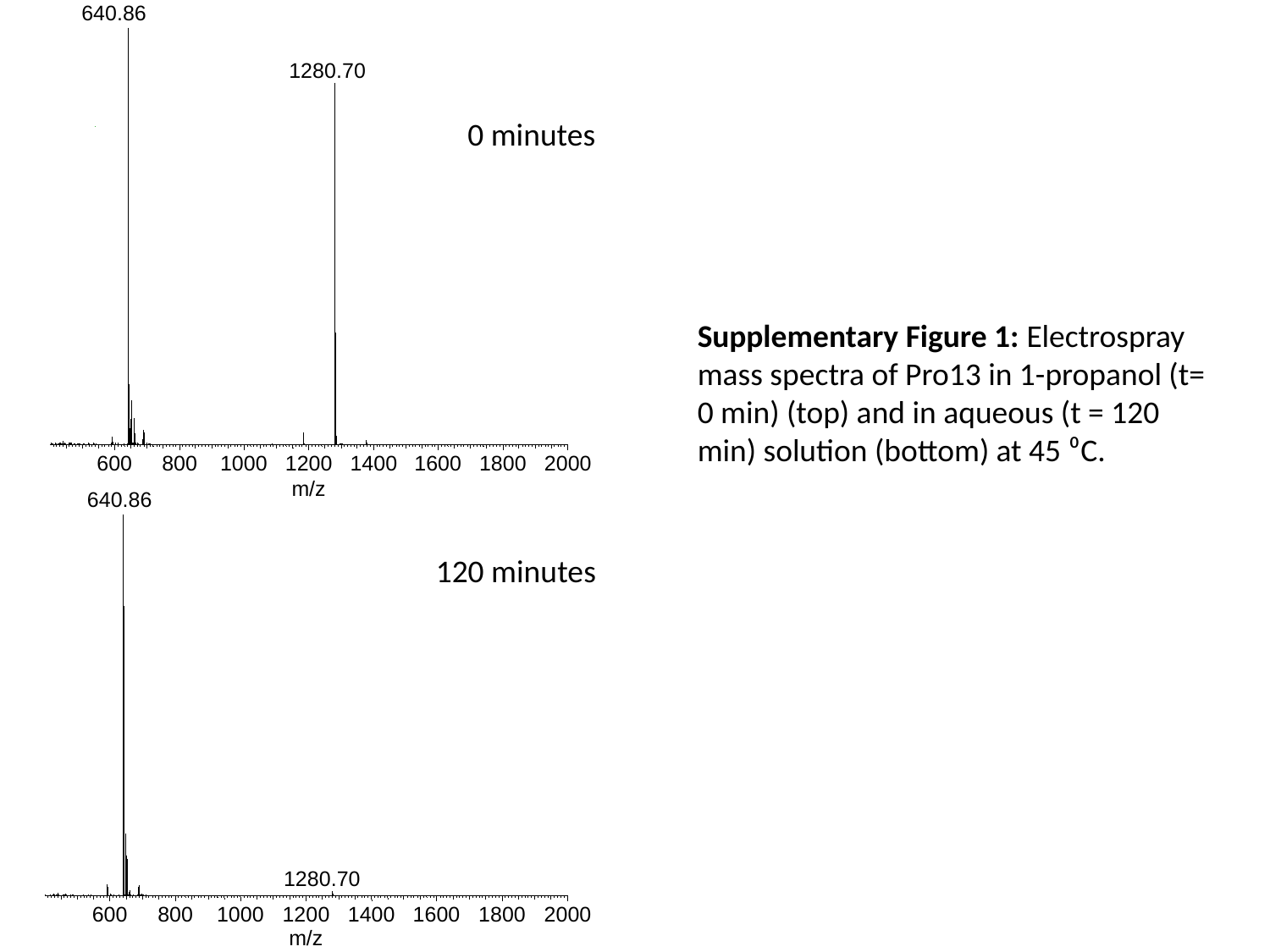

640.86
1280.70
600
800
1000
1200
1400
1600
1800
2000
m/z
0 minutes
Supplementary Figure 1: Electrospray mass spectra of Pro13 in 1-propanol (t= 0 min) (top) and in aqueous (t = 120 min) solution (bottom) at 45 ⁰C.
640.86
1280.70
600
800
1000
1200
1400
1600
1800
2000
m/z
120 minutes

## Slide 2
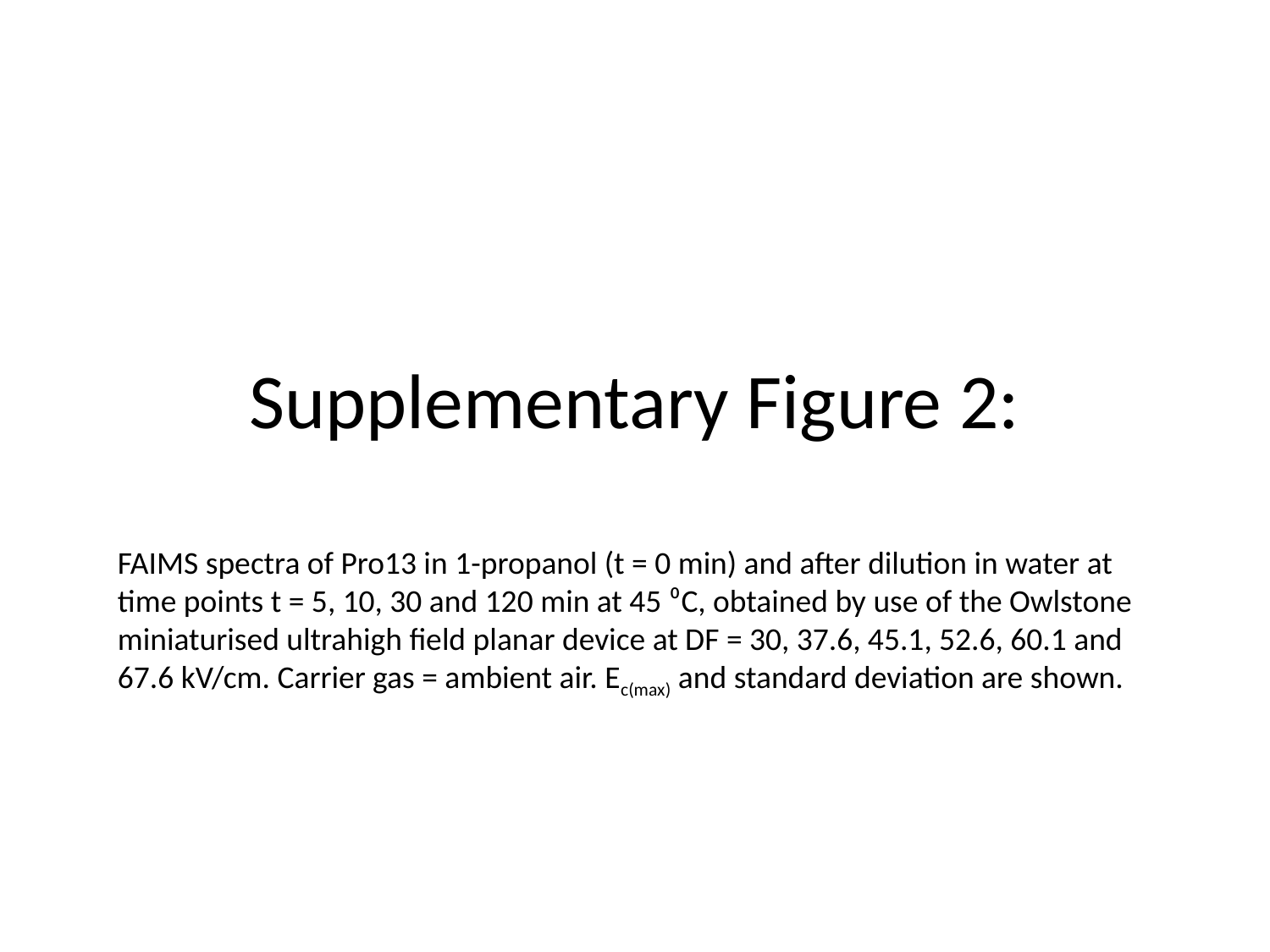

# Supplementary Figure 2:
FAIMS spectra of Pro13 in 1-propanol (t = 0 min) and after dilution in water at time points t = 5, 10, 30 and 120 min at 45 ⁰C, obtained by use of the Owlstone miniaturised ultrahigh field planar device at DF = 30, 37.6, 45.1, 52.6, 60.1 and 67.6 kV/cm. Carrier gas = ambient air. Ec(max) and standard deviation are shown.

## Slide 3
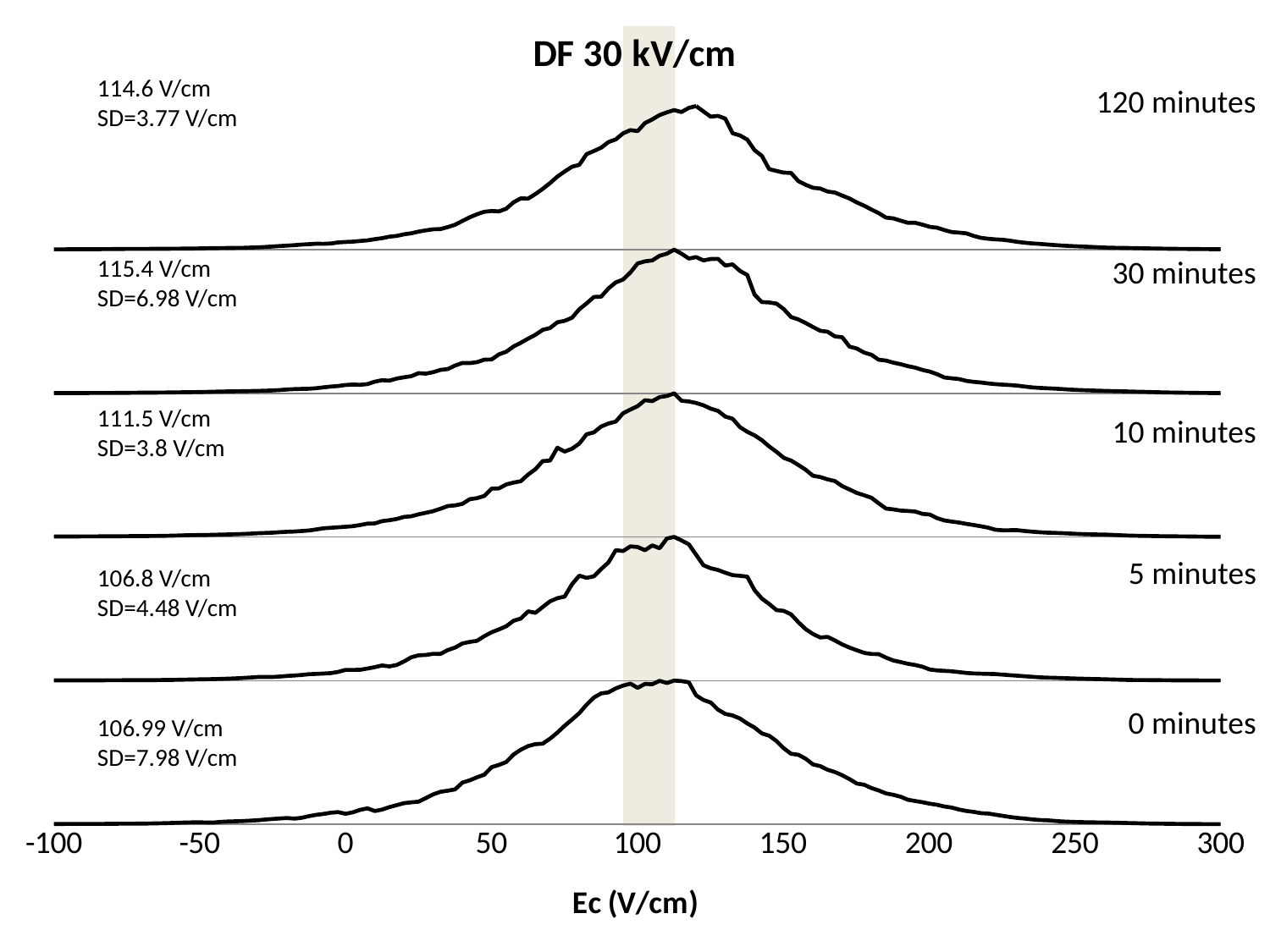

### Chart: DF 30 kV/cm
| Category | 0 min | 5 min | 10 min | 30 min | 120 min |
|---|---|---|---|---|---|
114.6 V/cm
SD=3.77 V/cm
120 minutes
115.4 V/cm
SD=6.98 V/cm
30 minutes
111.5 V/cm
SD=3.8 V/cm
10 minutes
5 minutes
106.8 V/cm
SD=4.48 V/cm
0 minutes
106.99 V/cm
SD=7.98 V/cm

## Slide 4
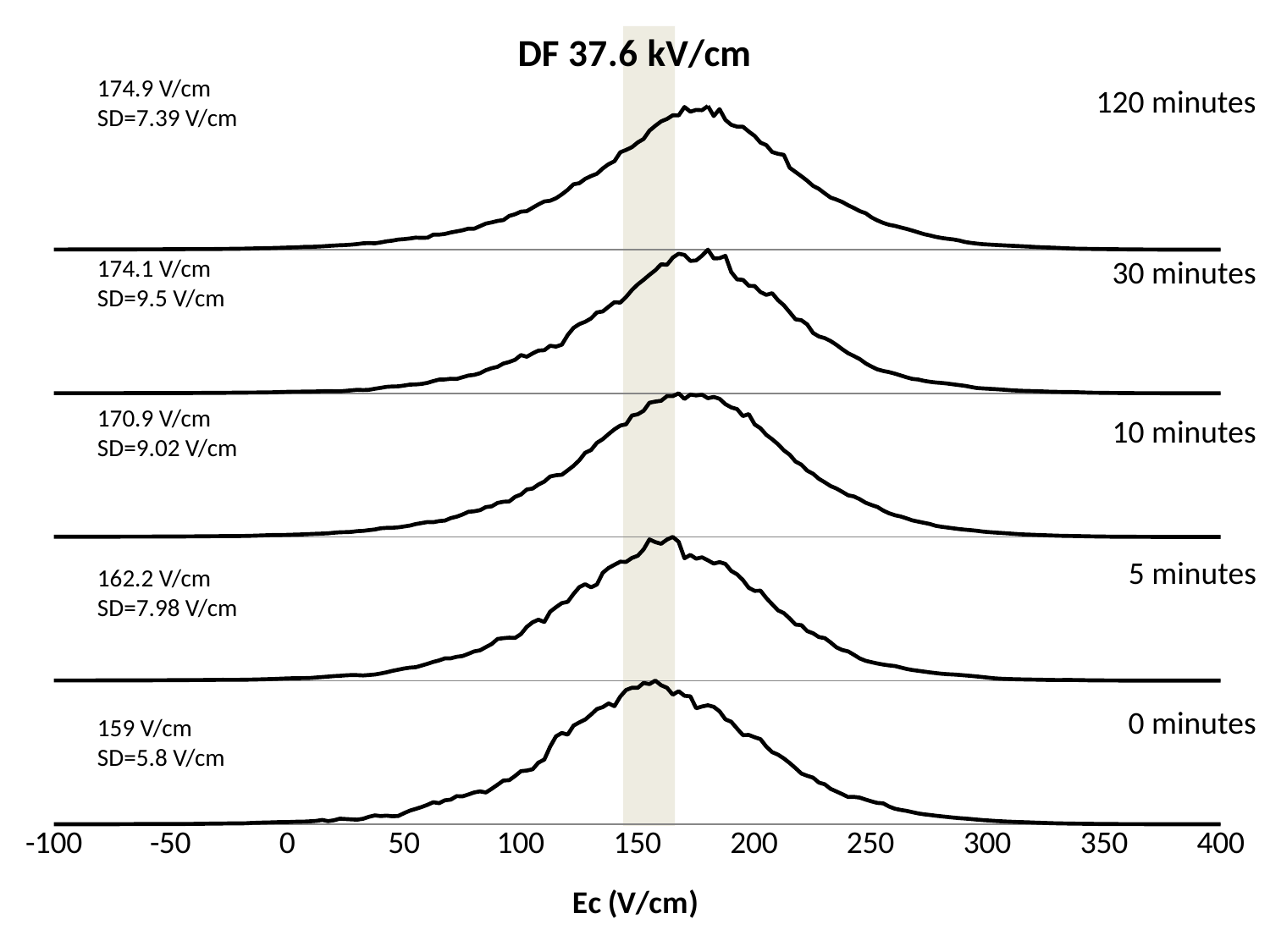

### Chart: DF 37.6 kV/cm
| Category | 0 min | 5 min | 10 min | 30 min | 120 min |
|---|---|---|---|---|---|
174.9 V/cm
SD=7.39 V/cm
120 minutes
174.1 V/cm
SD=9.5 V/cm
30 minutes
170.9 V/cm
SD=9.02 V/cm
10 minutes
5 minutes
162.2 V/cm
SD=7.98 V/cm
0 minutes
159 V/cm
SD=5.8 V/cm

## Slide 5
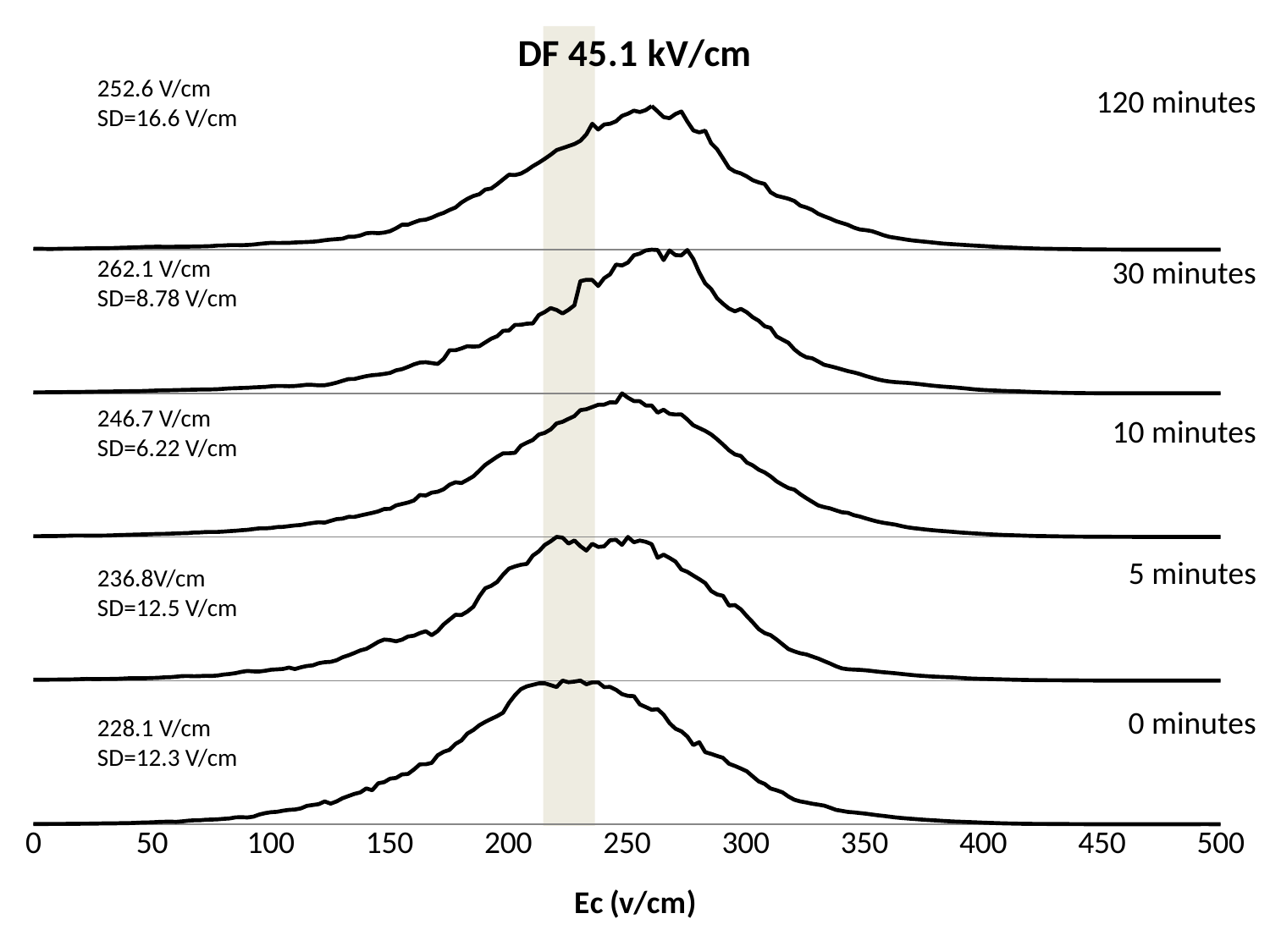

### Chart: DF 45.1 kV/cm
| Category | 0 min | 5 min | 10 min | 30 min | 120 min |
|---|---|---|---|---|---|
252.6 V/cm
SD=16.6 V/cm
120 minutes
262.1 V/cm
SD=8.78 V/cm
30 minutes
246.7 V/cm
SD=6.22 V/cm
10 minutes
5 minutes
236.8V/cm
SD=12.5 V/cm
0 minutes
228.1 V/cm
SD=12.3 V/cm

## Slide 6
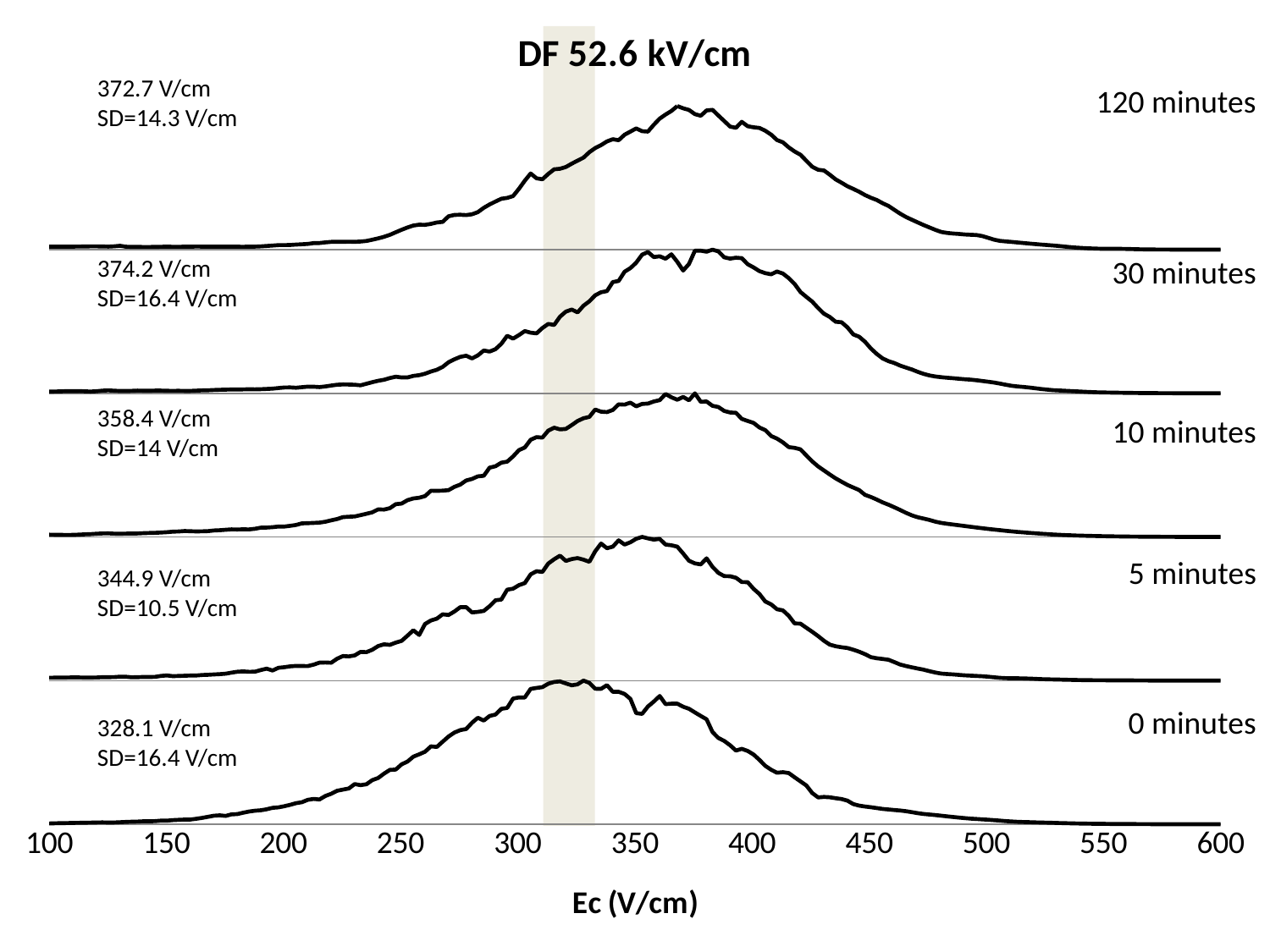

### Chart: DF 52.6 kV/cm
| Category | 0 min | 5 min | 10 min | 30 min | 120 min |
|---|---|---|---|---|---|
372.7 V/cm
SD=14.3 V/cm
120 minutes
374.2 V/cm
SD=16.4 V/cm
30 minutes
358.4 V/cm
SD=14 V/cm
10 minutes
5 minutes
344.9 V/cm
SD=10.5 V/cm
0 minutes
328.1 V/cm
SD=16.4 V/cm

## Slide 7
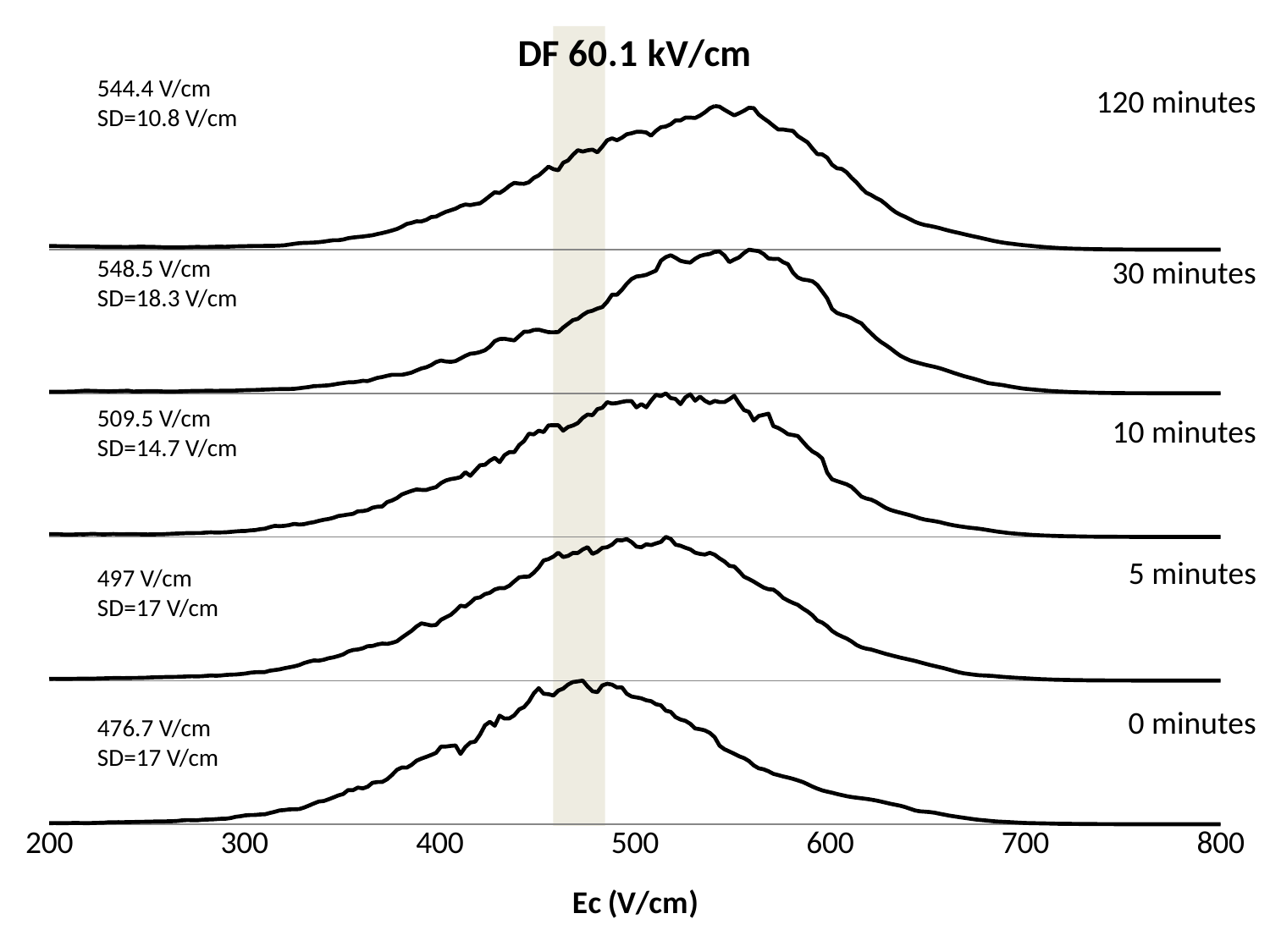

### Chart: DF 60.1 kV/cm
| Category | 0 min | 5 min | 10 min | 30 min | 120 min |
|---|---|---|---|---|---|
544.4 V/cm
SD=10.8 V/cm
120 minutes
548.5 V/cm
SD=18.3 V/cm
30 minutes
509.5 V/cm
SD=14.7 V/cm
10 minutes
5 minutes
497 V/cm
SD=17 V/cm
0 minutes
476.7 V/cm
SD=17 V/cm

## Slide 8
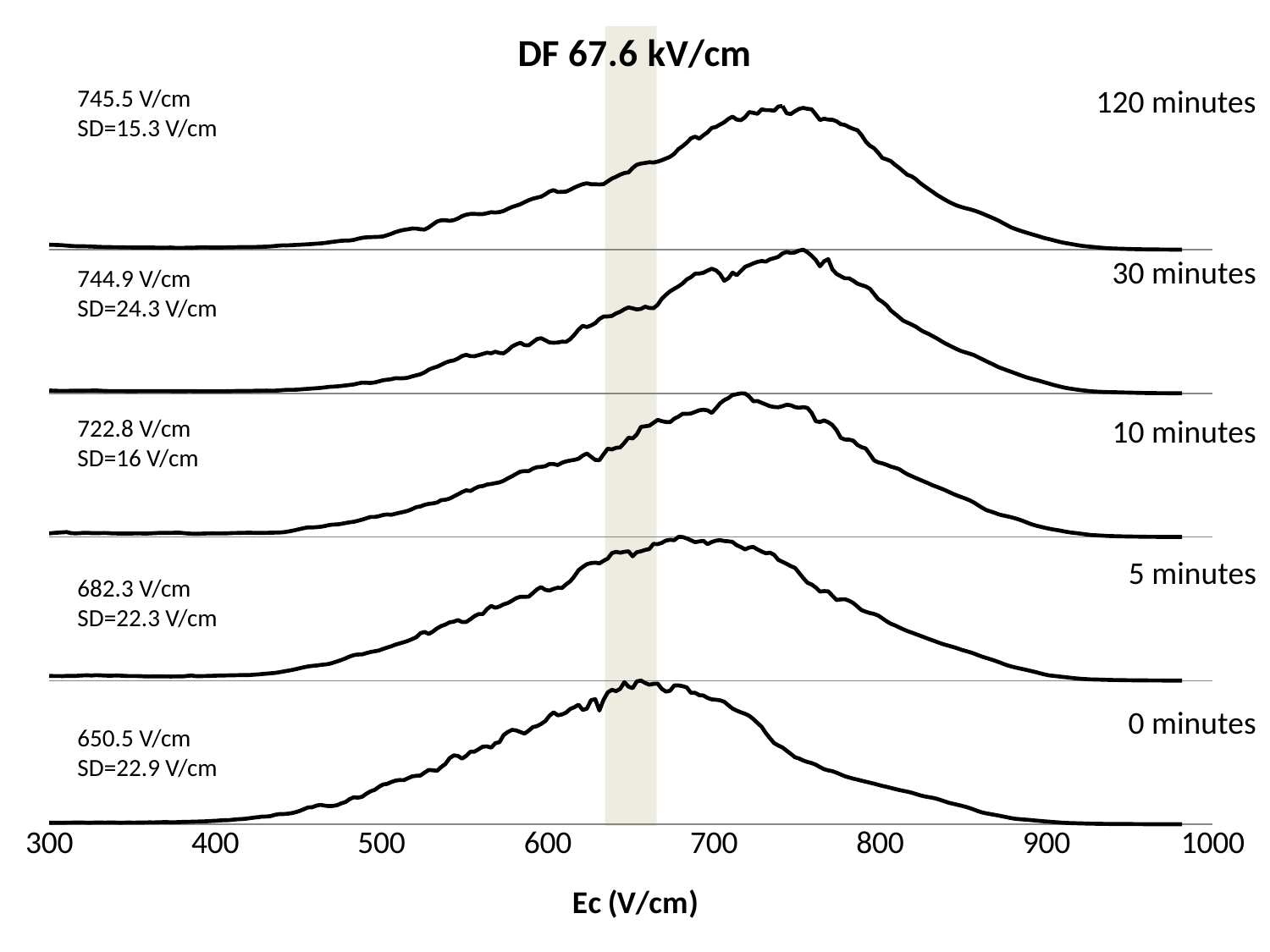

### Chart: DF 67.6 kV/cm
| Category | 0 min | 5 min | 10 min | 30 min | 120 min |
|---|---|---|---|---|---|
120 minutes
745.5 V/cm
SD=15.3 V/cm
30 minutes
744.9 V/cm
SD=24.3 V/cm
10 minutes
722.8 V/cm
SD=16 V/cm
5 minutes
682.3 V/cm
SD=22.3 V/cm
0 minutes
650.5 V/cm
SD=22.9 V/cm

## Slide 9
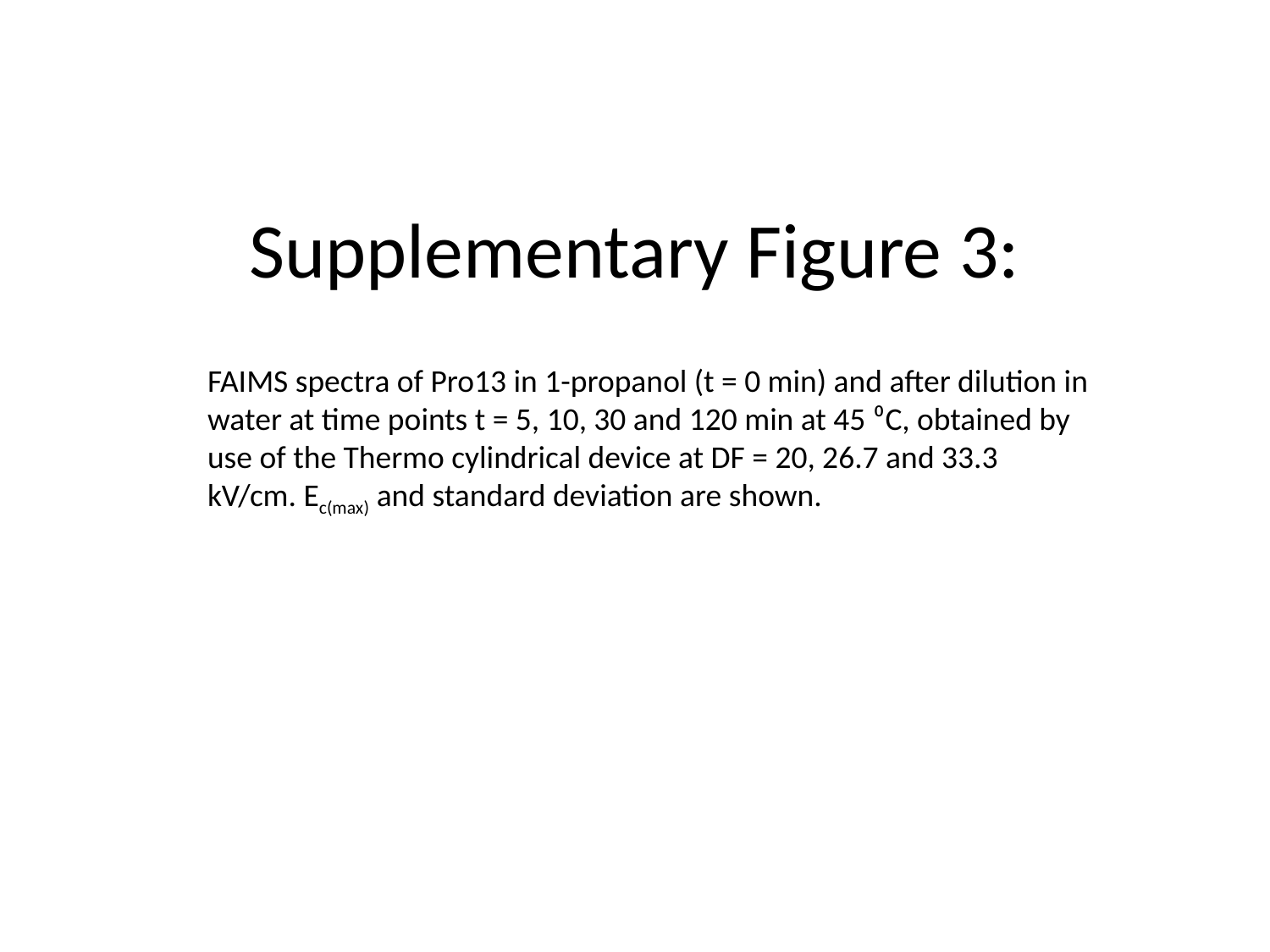

# Supplementary Figure 3:
FAIMS spectra of Pro13 in 1-propanol (t = 0 min) and after dilution in water at time points t = 5, 10, 30 and 120 min at 45 ⁰C, obtained by use of the Thermo cylindrical device at DF = 20, 26.7 and 33.3 kV/cm. Ec(max) and standard deviation are shown.

## Slide 10
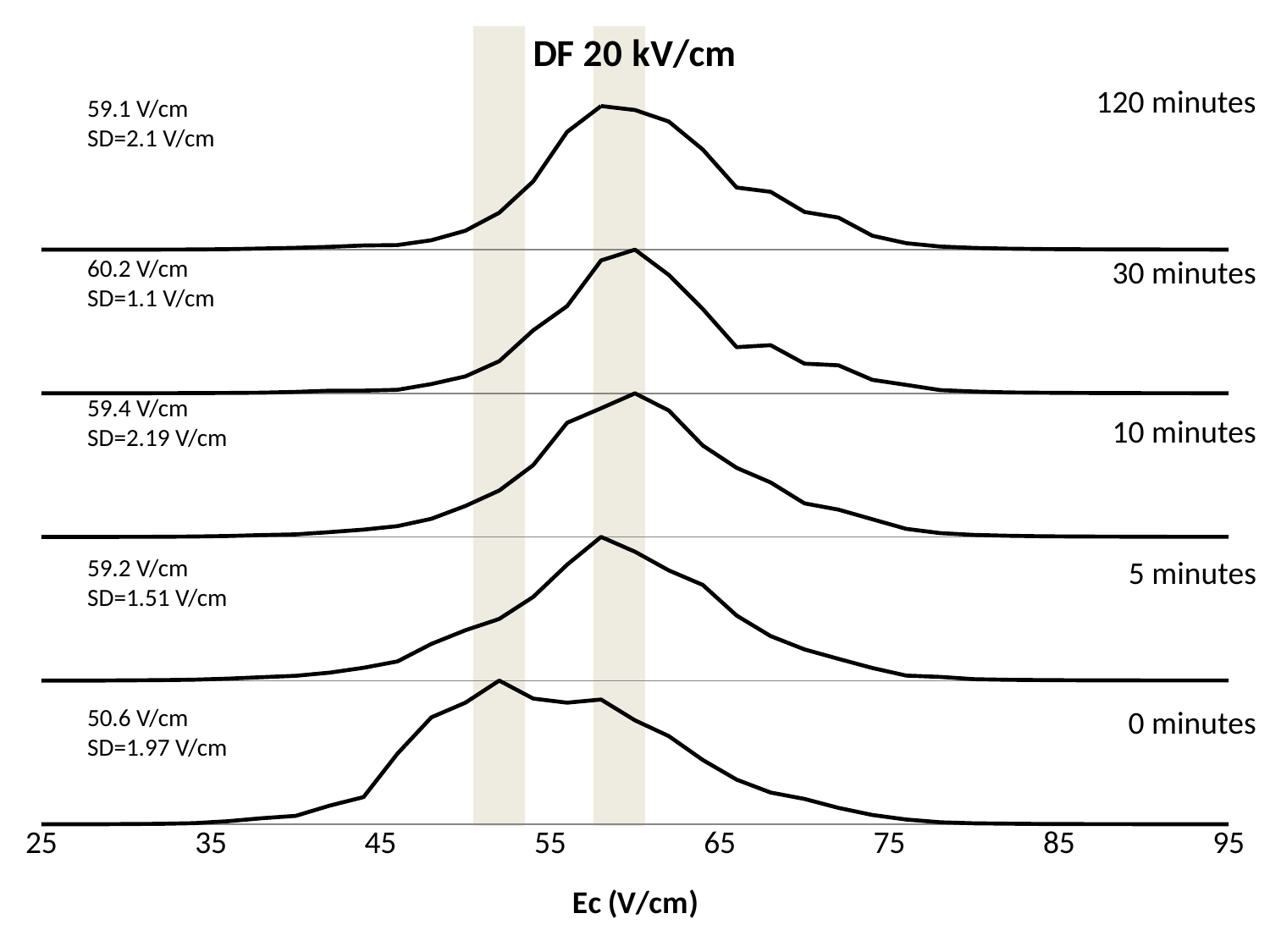

### Chart: DF 20 kV/cm
| Category | 0 min | 5 min | 10 min | 30 min | 120 min |
|---|---|---|---|---|---|
120 minutes
59.1 V/cm
SD=2.1 V/cm
60.2 V/cm
SD=1.1 V/cm
30 minutes
59.4 V/cm
SD=2.19 V/cm
10 minutes
59.2 V/cm
SD=1.51 V/cm
5 minutes
50.6 V/cm
SD=1.97 V/cm
0 minutes

## Slide 11
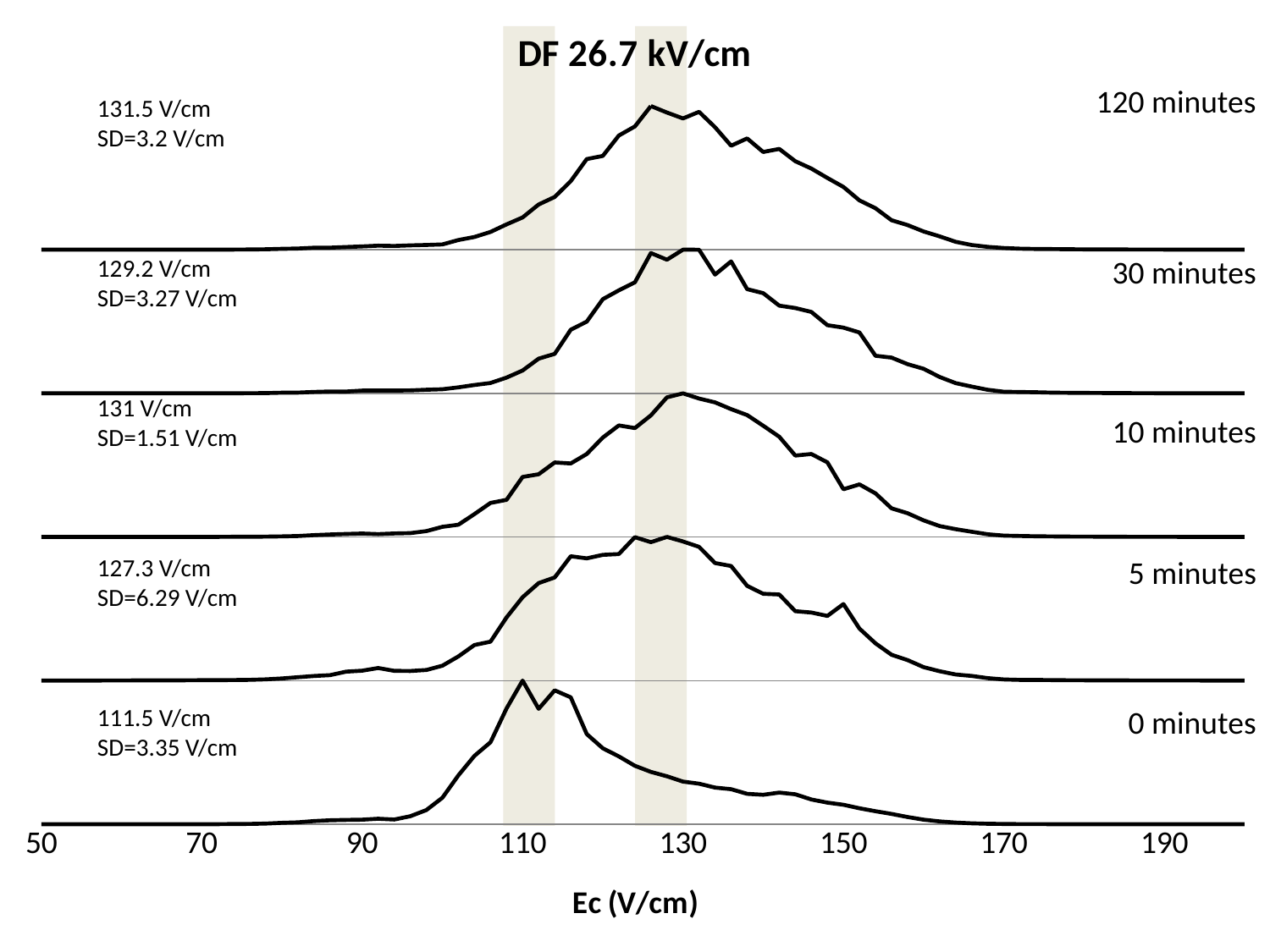

### Chart: DF 26.7 kV/cm
| Category | 0 | 5 min | 10 min | 30 min | 120 min |
|---|---|---|---|---|---|
120 minutes
131.5 V/cm
SD=3.2 V/cm
129.2 V/cm
SD=3.27 V/cm
30 minutes
131 V/cm
SD=1.51 V/cm
10 minutes
127.3 V/cm
SD=6.29 V/cm
5 minutes
111.5 V/cm
SD=3.35 V/cm
0 minutes

## Slide 12
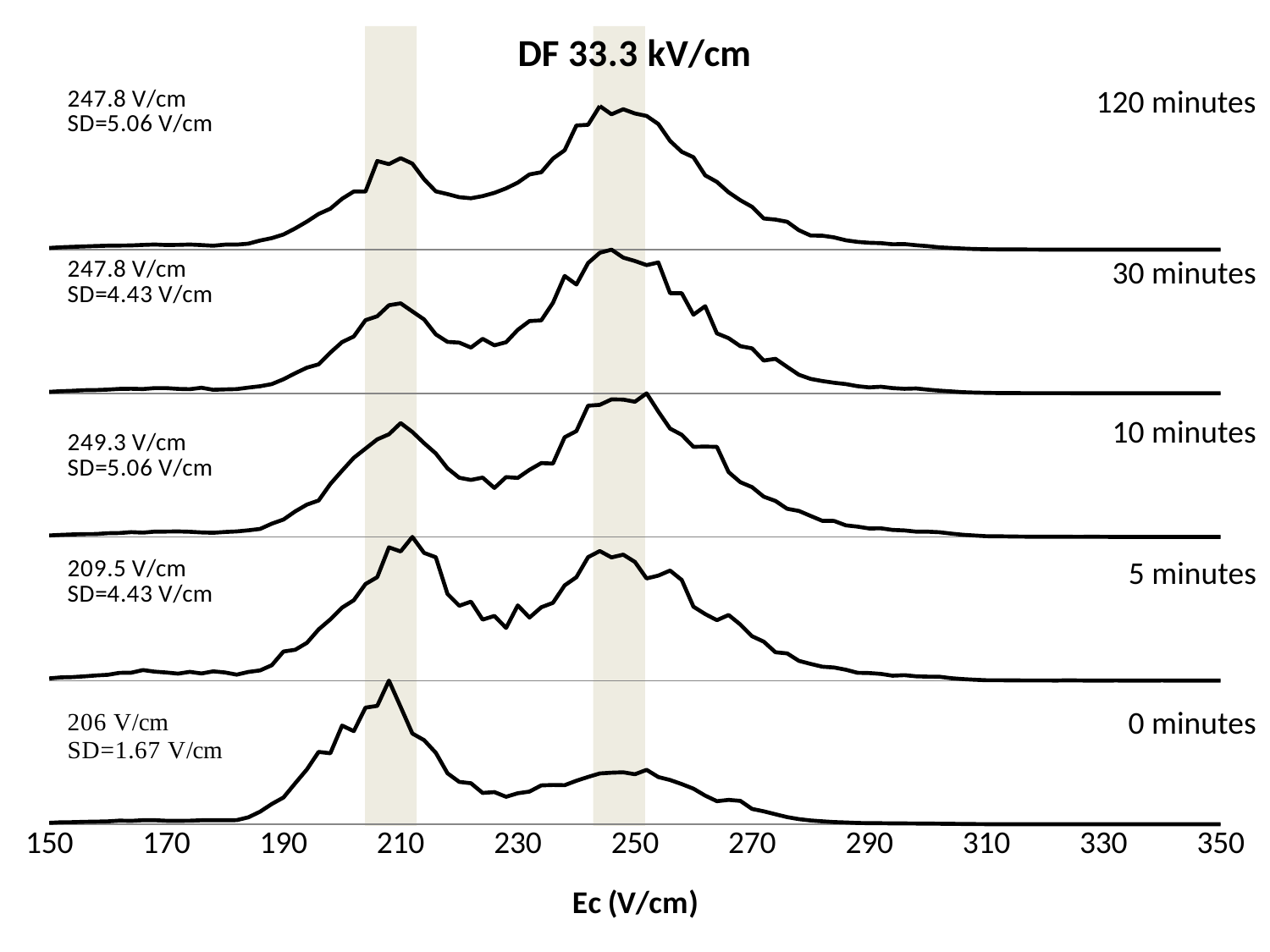

### Chart: DF 33.3 kV/cm
| Category | 0 min | 5 min | 10 min | 30 min | 120 min |
|---|---|---|---|---|---|
120 minutes
30 minutes
10 minutes
5 minutes
0 minutes
